# Poisonous Hair Dyes

**Published:** 1870-10

**Authors:** 


### POISONOUS HAIR DYES.

We have repeatedly warned our readers against patent dressings for the hair—so-called "hair restorers." They all depend upon the chemical action of lead and sulpher, upon the surface of the hair, for their coloring qualities, Lead, when taken into the system, is highly poisonous, giving rise to neuralgic affections and paralysis. When these nostrums are applied to the hair they come in contact with the scalp and are then absorbed and conveyed into the circulation giving rise to the affections above mentioned. Below we give the report of PROF. C. F. CHANDLER, who was employed by the New York Board of Health to analyze the different "hair tonics" with which the market is flooded. It will be seen that of the sixteen preparations which he examined, fifteen contained lead in varying proportions:

GRAINS OF LEAD IN ONE FLUID OUNCE.

| | | |
|---|---|---|
| 1. | Clark's Distilled Restorative for the Hair | 0.11 |
| 2. | Chevalier's Life for the Hair. | 1.02 |
| 3. | Circassian Hair Rejuvinator. | 2.71 |
| 4. | Ayer's Hair Vigor. | 2.89 |
| 5. | Prof. Wood's Hair Restorative. | 3.08 |
| 6. | Dr. J. J. O'Brien's Hair Restorer of America. | 3.28 |
| 7. | Gray's Celebrated Hair Restorative. | 3.39 |
| 8. | Phalon's Vitalia. | 4.69 |
| 9. | Ring's *Vegetable* Ambrosia. | 5.09 |
| 10. | Mrs. S. A. Allen's World's Hair Restorer. | 5.57 |
| 11. | L. Knittel's Indian Hair Tonic. | 6.29 |
| 12. | Hall's *Vegetable* Sicilian Hair Renew'r | 7.13 |
| 13 | .Dr. Tibbet's Physiological Hair Regenerator. | 7.44 |
| | Martha Washington's Hair Restor'tve. | 9.80 |
| [illegible]5. | Singer's Hair Restorative. | 16.39 |

We hope, after the above exhibit, our lady and bachelor friends will find it profitable to abandon the use of so-called "hair restorers."

